# Enhanced Recovery after Pediatric Cardiac Surgery: A Meta-Analysis

**DOI:** 10.1055/s-0045-1808072

**Published:** 2025-05-06

**Authors:** Osama Abu-Shawer, Abdel-Rahman E'mar, Abdel-Rahman Jaber, Shatha Tailakh, Amer Abu-Shawer, Caroline Al-Haddadin

**Affiliations:** 1 Anesthesiology and Perioperative Medicine, Case Western Reserve University, University Hospitals Cleveland Medical Center, Cleveland, Ohio, United States; 2 Departement of Pediatrics, Cleveland Clinic Foundation, Cleveland, Ohio, United States; 3 School of Medicine, University of Jordan, Amman, Jordan

**Keywords:** enhanced recovery, ERAS, pediatric cardiac surgery

## Abstract

**Background:**

The Enhanced Recovery After Surgery (ERAS) protocols are a set of steps taken before, during, and after surgery to improve patient care and outcomes. While ERAS is well known for its benefits in various surgeries, its application in pediatric cardiac surgery is relatively new. With the recent emergence of studies on its implementation in pediatric cardiac surgery, this study is the first to systematically review the current evidence on the efficacy of ERAS in the field.

**Methods:**

A meta-analysis was performed according to Preferred Reporting Items for Systematic Reviews and Meta-Analyses (PRISMA) guidelines. Two reviewers independently searched PubMed, Cochrane, Google Scholar, Web of Science, Embase, and Scopus databases for comparative studies with control groups that described the use of ERAS in all types of pediatric cardiac surgeries from 2000 to 2024. The data collected included study design, patient demographics, elements of the ERAS protocols, and postoperative outcomes. The random-effects model was used to calculate the pooled odds ratios (ORs) and mean differences (MDs) with the corresponding confidence intervals (CIs) for proportional and continuous variables, respectively.

**Results:**

Five studies, involving 1,008 patients, were included in the final analysis: three randomized controlled trials (RCTs), one retrospective cohort, and one case-control study. The ERAS protocols were applied in 430 (43%) patients, and standard perioperative care was applied in 578 (57%) patients. The analysis revealed that implementing the ERAS protocol significantly reduced ICU length of stay ( *I* ^2^ = 98.26%; MD = −1.441; 95% CI: −2.610 to −0.273; *p* = 0.016). The ERAS group had a comparable rate of postoperative complications to the standard care group ( *I* ^2^ = 15.3%; OR: 0.889; 95% CI: 0.622–1.269; *p* = 0.516).

**Conclusions:**

The ERAS protocols in pediatric cardiac surgery appear to be safe and effective in improving certain short-term outcomes. However, evidence is limited due to the small number of studies. Further multicenter RCTs that fully incorporate the ERAS protocol elements and assess both immediate and long-term outcomes are needed.

## Introduction and Background

The Enhanced Recovery After Surgery (ERAS) protocol is a comprehensive approach that includes preoperative, intraoperative, and postoperative care strategies to accelerate recovery. 1 While the ERAS protocol has been well documented in adult cardiac surgery as an effective method to improve clinical outcomes and reduce postoperative complications, some studies have also demonstrated its benefits in pediatric cardiac surgery. These benefits include shorter stays in the intensive care unit (ICU), reduced use of blood products, lower levels of postoperative pain, and fewer postoperative complications. 2 3 4 This study aims to systematically analyze the effects of the ERAS protocol in pediatric cardiac surgery, focusing on its impact on the ICU length of stay (LOS), mortality, postoperative complications, and readmission rates.

This meta-analysis was conducted in accordance with the Preferred Reporting Items for Systematic Reviews and Meta-Analyses (PRISMA) guidelines. Since our study involved the statistical analysis of already published data, neither ethical approval nor informed consent was necessary.

### Search Strategy

We conducted a comprehensive systematic review by searching several databases, including Medline (via Ovid SP), Embase (via Ovid SP), Web of Science (via Clarivate Analytics), Scopus (via Elsevier), CINHAL (via EBSCOhost), and Cochrane (via Wiley). Our aim was to compare the effects of the ERAS and traditional care (TC) protocols on postoperative outcomes using a random-effects model.

The literature search covered the period from 2000 to 2024, employing keywords such as “enhanced recovery after surgery,” “child,” “postoperative complication,” “length of stay,” “hospital readmission,” “in-hospital mortality,” “hospitalization cost,” and “treatment.” Both controlled vocabulary and free-text terms were utilized. Detailed search strategies for all databases are provided in Supplementary Tables S1 – S6 (available in the online version only). Articles were imported into EndNote and then transferred to Covidence, where they were screened for duplicates.

### Study Selection

Two independent reviewers evaluated the systematically searched titles and abstracts using a standardized, pretested form. References from studies meeting the inclusion criteria were manually reviewed to ensure that all pertinent articles were captured. Any discrepancies during the full-text screening were resolved through mutual agreement between the reviewers.

### Inclusion Criteria

Eligible studies included randomized controlled trials (RCTs), as well as prospective and retrospective cohort studies or case-control studies. The research must involve pediatric patients (18 years or younger) undergoing cardiac surgery and compare the ERAS protocols with standard postoperative care. Studies should report at least one of the predefined outcomes relevant to this meta-analysis (outlined below).

### Exclusion Criteria

Studies such as meta-analyses, systematic reviews, case reports, case series, letters, surveys, editorials, or conference abstracts were excluded. Noncomparative studies were also excluded, as well as studies not involving cardiac surgery patients or those including adult participants (older than 18 years). Additionally, studies that did not report any of the predefined outcomes for this meta-analysis were not considered.

## Outcomes

This meta-analysis examined the postoperative mortality within the included studies, the postoperative LOS measured as the number of days from the initial surgery to the patient's discharge, the 30-day readmission defined as a hospital readmission within 30 days of the initial surgery, and the incidence of postoperative complications such as infections, acute kidney injury, bleeding, and respiratory failure.

### Statistical Analysis

We performed a meta-analysis of the selected studies using Review Manager 5.3 and Comprehensive Meta-Analysis 3.3 software. When required, medians and interquartile ranges were converted to means and standard deviations. 5 A random-effects model was utilized to calculate odds ratios (ORs) for categorical variables and mean differences (MDs) for continuous variables, along with their respective confidence intervals (CIs). 6 Statistical significance was defined as a *p-* value of ≤0.05. Heterogeneity among studies was evaluated using the *I* ^2^ statistic in accordance with the Cochrane Handbook for Systematic Reviews. An *I* ^2^ value of ≥50% was considered indicative of significant heterogeneity for all outcomes. 6

### Review

**Fig. 1:**
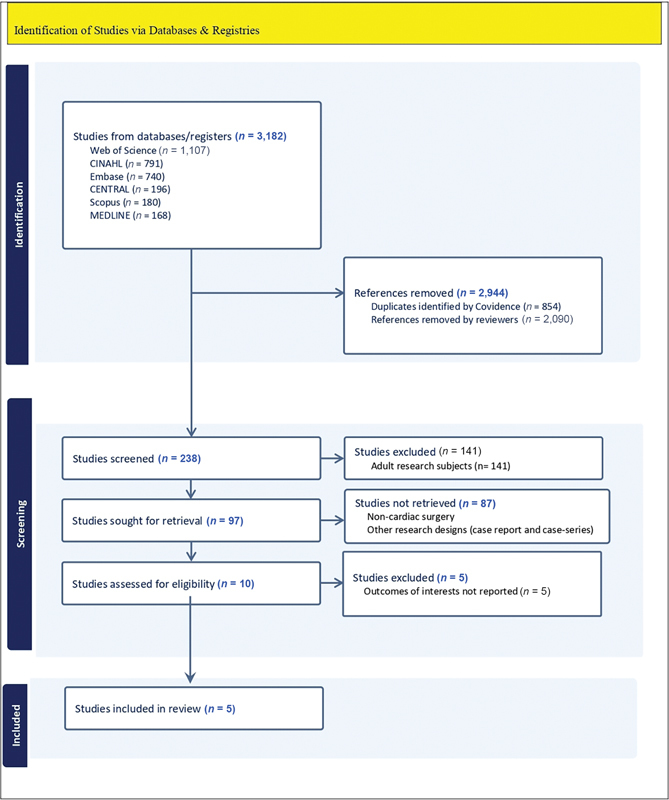
Identification of studies via databases and registries.

Using Covidence software, we retrieved 3,182 records from the searched databases. After removing 854 duplicate records identified by the software, the reviewers excluded an additional 2,090 articles based on their titles and abstracts. This left 238 articles for eligibility screening. Of these, 141 were excluded due to the study population age, and 87 were excluded based on the type of surgery and study design. The full texts of the remaining 10 studies were reviewed for final eligibility, resulting in the exclusion of 5 studies due to unsuitable outcomes. Therefore, five studies were ultimately included in this meta-analysis. 7 8 9 10 11 The literature search and screening process is depicted in Fig. 1 .

The five studies included in the meta-analysis were published between 2009 and 2024. The sample sizes of the individual studies ranged from 72 to 452, with a total of 1,008 participants. Of the five studies, three were randomized clinical trials, one was a cohort study, and one was a case-control study. The baseline characteristics of the included studies are presented in Table 1 .

**Table 1:** Baseline characteristics of the included studies

Study	Institute	Journal	Design	Included populations	Sample size (total)	ERAS	Non-ERAS
Roy et al 7	Boston Children's Hospital	Journal of Thoracic and Cardiovascular Surgery	Cohort	Cardiac surgery	452	151	301
Andugala, et al 8	Queensland Children's Hospital	Heart, Lung and Circulation	Case control	Cardiac surgery	190	95	95
Abdelbaser and Mageed 8	Mansoura University	Journal of Clinical Anesthesia	RCT	Cardiac surgery	73	37	36
Xu et al 10	China General Hospital	BMC Pediatrics	RCT	Cardiac surgery	194	97	97
Preisman et al 11	Sheba Medical Center	Journal of Cardiothoracic and Vascular Anesthesia	RCT	Cardiac surgery	99	50	49

Abbreviation: ERAS, Enhanced Recovery After Surgery; RCT, randomized controlled trial.

Of the 1,008 patients undergoing cardiac surgery, 430 (43%) received the ERAS protocols, while 578 (57%) received the standard postoperative care. The baseline characteristics of these patients are detailed in Table 2 . A study reporting mortality rates found no significant difference between the ERAS and TC groups ( Table 2 ).

**Table 2:** Baseline characteristics of the included population

Study	Age (ERAS)	Age (Non-ERAS)	Gender, M/F (ERAS)	Gender, M/F (Non-ERAS)	Mortality (ERAS)	Mortality (Non-ERAS)	Readmission (ERAS)	Readmission (Non-ERAS)
Roy et al 7	3.8 (0.5–12.3)	3.3 (0.5–9.4)	79/72	168/133	NR	NR	7 (4.6%)	20 (7.7%)
Andugala et al 8	4.3 (2.7–6.8)	4.4 (1.9–7.6)	45/50	32/63	NR	NR	5 (5.3%)	3 (3.2%)
Abdelbaser and Mageed 8	7 (2–12)	5 (2–12)	19/18	19/17	NR	NR	NR	NR
Xu et al 10	1.2 (0.7–1.7)	1.1 (0.6–1.6)	45/52	49/48	NR	NR	NR	NR
Preisman et al 11	1.92 (0.17–15.0)	0.92 (0.08–11.0)	NR	NR	2 (4%)	2 (4%)	NR	NR
	18.2 (0.17–15.0)	14.7 (0.08–11.0)	188/142	268/261	2 (4%)	2 (4%)	12 (4.8%)	23 (7.7%)

Abbreviation: ERAS, Enhanced Recovery After Surgery; NR, not reported.

### Meta-Analysis of the Length of ICU Stay

All of the included studies reported on the ICU LOS. The meta-analysis demonstrated that the ERAS protocol significantly shortened the ICU stay compared to the TC protocol ( *I* ^2^ = 98.26%; MD = −1.441 days (∼34 hours); 95% CI = −2.610 to −0.273; *p* = 0.016). A summary of the findings from the included studies is presented in Table 3 .

**Table 3:** The length of ICU stay results

Study	ERAS			Non-ERAS			Standard difference in means	Standard error	Difference in means	Standard error
	Mean (d)	SD	Total	Mean (d)	SD	Total				
Roy et al 7	1.366	0.808	151	1.452	0.842	301	−0.104	0.100	−0.086	0.083
Andugala et al 8	0.218	0.113	95	0.965	0.226	95	−4.181	0.259	−0.747	0.026
Abdelbaser and Mageed 8	1.089	0.054	37	1.335	0.226	36	−1.507	0.265	−0.246	0.038
Xu et al 10	0.800	0.452	97	1.165	0.226	97	−1.021	0.153	−0.365	0.051
Preisman et al 11	4.068	6.641	50	9.337	14.741	49	−0.462	0.204	−5.269	2.290
	1.275	2.555	430	1.985	4.849	578	−1.441	0.596	−1.342	0.497

Abbreviation: ERAS, Enhanced Recovery After Surgery; ICU, intensive care unit; SD, standard deviation.

### Meta-Analysis of the Rate of Complications

All included studies reported postoperative complication rates. The meta-analysis showed no significant difference between the ERAS and TC protocols in terms of complication rates ( *I* ^2^ = 15.3%; OR = 0.889; 95% CI = 0.622–1.269; *p* = 0.516). The results are summarized in Table 4 .

**Table 4:** The postoperative complication results

Study	ERAS		Non-ERAS		Odds ratio (OR)	Log OR	Standard error
	Events, *n* (%)	Total	Events, *n* (%)	Total			
Roy et al 7	25 (16.5%)	151	52 (17%)	301	0.950	−0.051	0.267
Andugala et al 8	13 (14%)	95	8 (8.4%)	95	1.724	0.545	0.475
Abdelbaser and Mageed 8	14 (38%)	37	14 (40%)	36	0.957	−0.044	0.481
Xu et al 10	3 (3%)	97	4 (4%)	97	0.742	−0.298	0.778
Preisman et al 11	16 (32%)	50	25 (51%)	49	0.452	−0.795	0.417

Abbreviation: ERAS, Enhanced Recovery After Surgery.

## Discussion

ERAS is a relatively new approach in surgery designed to improve clinical outcomes and reduce health care costs. 12 Recent studies have shown growing interest in applying the ERAS protocol to pediatric surgical patients, demonstrating its applicability and safety across various surgical settings. 13 14 15 16

The benefits of ERAS are well documented in the literature, including reductions in the hospital LOS, 30-day readmission rates, and hospitalization-related costs. 17 18 19 20 21 In our study, the ICU LOS was significantly reduced in the ERAS group, although the 30-day readmission rate, evaluated in only two studies, showed no difference between the ERAS and TC groups. Recent research indicates that the ERAS protocol does not significantly alter complication rates, which our results support, showing no significant difference in postoperative complications between the groups. 13 14 Previous studies have reported reduced postoperative mortality rates with ERAS implementation, but our analysis found similar mortality rates between the ERAS and TC groups, based on one study. 20 21

Our study has several limitations. The small number of included studies may limit the generalizability of our findings. Additionally, only one study reported on postoperative mortality, and there is a lack of data on other relevant outcomes, such as opioid use, timing of oral nutrition, timing of mobilization, and hospitalization-related costs.

## Conclusions

This meta-analysis evaluated the efficacy and safety of ERAS compared to TC protocols in pediatric patients undergoing cardiac surgery. A total of five studies, encompassing 1,008 patients, were included in the analysis. Our findings indicate a significant reduction in the ICU LOS for patients following the ERAS protocol compared to those in the TC group. Additionally, the postoperative complication rates were similar between the ERAS and standard perioperative protocol groups.

The application of the ERAS protocol in pediatric cardiac surgery appears effective in reducing the ICU LOS and maintaining similar postoperative complication rates compared to TC. However, more RCTs are needed to rigorously assess the ERAS protocol and explore its impact on a wider range of clinical outcomes in pediatric cardiac surgery.
