# Supplementary material for: Enhanced Recovery after Pediatric Cardiac Surgery: A Meta-Analysis
Source: Avicenna J Med. 2025 May 6;15(2):80–5. doi: 10.1055/s-0045-1808072 (PMC12178667; doi:10.1055/s-0045-1808072)
Supplement: Supplementary file 1 — Supplementary Material [file 10-1055-s-0045-1808072-s240149.pdf]

Supplementary Table S1 Embase, 2000 to April 3, 2024

1	enhanced recovery after surgery/ or (“enhanced post operative recovery” or “enhanced postoperative recovery” or “enhanced recovery after surgery” or “enhanced recovery”).mp.	13,668
2	exp child/ or exp “congenital, hereditary, and neonatal diseases and abnormalities”/ or exp infant/ or exp adolescence/ or exp infant, newborn/ or exp child, preschool/ or (pediatric* or paediatric* or child* or newborn* or congenital* or infan* or baby or babies or neonat* or pre-term or premature birth or NICU or preschool* or preschool* or kindergarten* or elementary school* or nursery school* or schoolchild* or toddler* or boy or boys or girl* or middle school* or pubescen* or juvenile* or teen* or youth* or high school* or adolesc* or pre-pubesc*).mp. or (child* or adolesc* or pediat* or paediat*).jn.	6,360,477
3	exp postoperative complication/ or ((post operative or post-operative or post-surg* or post surg* or surg*) adj2 complication*).mp.	889,119
4	exp “length of stay”/ or “length of stay”.mp. or hospital stay*.mp. or stay length*.mp. [mp = title, abstract, heading word, drug trade name, original title, device manufacturer, drug manufacturer, device trade name, keyword heading word, floating subheading word, candidate term word]	427,710
5	exp hospital readmission/ or “patient readmission”.mp. or 30 day readmission.mp. or readmission.mp. or rehospitalization.mp. or rehospitalisation.mp. [mp = title, abstract, heading word, drug trade name, original title, device manufacturer, drug manufacturer, device trade name, keyword heading word, floating subheading word, candidate term word]	117,708
6	in-hospital mortality.mp. or in-hospital mortality/ or ((inhospital or in-hospital or hospital or in-house or in house) adj2 (death or mortalit*)).mp. [mp = title, abstract, heading word, drug trade name, original title, device manufacturer, drug manufacturer, device trade name, keyword heading word, floating subheading word, candidate term word]	134,067
7	“hospitalization cost”/ or ((hospitalisation or hospitalization or hospital*) adj2 cost*).mp. [mp = title, abstract, heading word, drug trade name, original title, device manufacturer, drug manufacturer, device trade name, keyword heading word, floating subheading word, candidate term word]	56,393
8	exp treatment outcome/	2,386,365
9	or/3-8	3,404,511
10	case report.mp.	3,029,505
11	1 and 2 and 9	778
12	11 not 10	740

Supplementary Table S2 MEDLINE ALL, 2000 to April 3, 2024

1	Enhanced Recovery After Surgery/ or (enhanced post operative recovery or enhanced post-operative recovery or enhanced postsurgical recovery or enhanced recovery after surgery or postsurgical recoveries, enhanced or postsurgical recovery, enhanced or recovery, enhanced postsurgical or enhanced recovery).mp.	7,771
2	exp *adolescent/ or exp *child/ or exp *infant/ or (infant disease* or childhood disease*).ti,kf. or (adolescen* or babies or baby or boy? or boyfriend or boyhood or child* or girl? or infant* or juvenil* or kid? or minors or minors* or neonat* or neonat* or newborn* or new-born* or paediatric* or peadiatric* or pediatric* or perinat* or preschool* or puber* or pubescen* or school* or teen* or toddler? or underage? or under-age? or youth*).ti,kf.	2,057,471
3	Postoperative Complications/ or ((post operative or post-operative or post-surg* or post surg* or surg*) adj2 complication*).mp.	445,555
4	“Length of Stay”/ or (hospital stay or hospital stays or “length of stay” or stay, hospital or stay length or stay lengths or stays, hospital).mp.	229,053
5	Patient Readmission/ or 30 day readmission.mp. or readmission.mp. or rehospitalization*.mp. or rehospitalisation*.mp.	49,707
6	exp Hospital Mortality/ or ((in-hospital or inhospital or in house or in-house) adj2 (death or mortalit*)).mp.	75,512
7	exp Hospital Costs/ or ((hospitalisation or hospitalization or hospital) adj2 cost*).mp.	29,026
8	treatment outcome/	1,181,484
9	or/3-8	1,767,262
10	1 and 2 and 9	169
11	case report.mp.	428,281
12	10 not 11	168

 © 2025. The Author(s).

Supplementary Table S3 Cochrane Library, 2000 to April 5, 2024

ID	Search	Hits
#1	MeSH descriptor: [Enhanced Recovery After Surgery] explode all trees	170
#2	(“enhanced post operative recovery” or “enhanced postoperative recovery” or “enhanced recovery after surgery” or “enhanced recovery”)	2,203
#3	(infant disease* or childhood disease*) or (adolescen* or babies or baby or boy? or boyfriend or boyhood or child* or girl? or infant* or juvenil* or kid? or minors or minors* or neonat* or neonat* or newborn* or new-born* or paediatric* or peadiatric* or pediatric* or perinat* or preschool* or puber* or pubescen* or school* or teen* or toddler? or underage? or under-age? or youth*).	491,521
#4	MeSH descriptor: [Child] explode all trees	81,699
#5	MeSH descriptor: [Postoperative Complications] explode all trees	54,926
#6	((post operative or post-operative or post-surg* or post surg* or surg*) NEAR/2 complication*)	21,225
#7	MeSH descriptor: [Length of Stay] explode all trees	9,501
#8	hospital stay or hospital stays or length of stay or stay length or stay lengths	53,932
#9	30 day readmission or readmission or rehospitalization* or rehospitalisation*	11,221
#10	MeSH descriptor: [Patient Admission] explode all trees	823
#11	MeSH descriptor: [Hospital Mortality] explode all trees	1,880
#12	((in-hospital or inhospital or in house or in-house) NEAR/2 (death or mortalit*))	38,858
#13	MeSH descriptor: [Hospital Costs] explode all trees	805
#14	((hospitalisation or hospitalization or hospital) NEAR/2 cost*)	5,672
#15	MeSH descriptor: [Treatment Outcome] explode all trees	201,115
#16	#1 OR #2	2,203
#17	#3 OR #4	491,521
#18	#5 OR #6	71,124
#19	#7 OR #8	53,932
#20	#9 OR #10	11,974
#21	#11 OR #12	39,754
#22	#13 OR #14	5,672
#23	{OR #18-#22, #15}	328,116
#24	#16 AND #17 AND #23	201
#25	5 Protocols removed	196

Supplementary Table S4 Cinahl (Ebsco interface): 2000 to April 5, 2024

S1 (MH “Enhanced Recovery After Surgery”) OR (enhanced post operative recovery or enhanced post-operative recovery or enhanced postsurgical recovery or enhanced recovery after surgery or postsurgical recoveries, enhanced or postsurgical recovery, enhanced or recovery, enhanced postsurgical or enhanced recovery)
S2 ((MH “Adolescence”) OR (MH “Child”) OR (MH “Child, Preschool”) OR (MH “Infant + ”) OR (MH “Infant, Newborn + ”)) OR ((infant disease* or childhood disease*) or adolescen* or babies or baby or boy? or boyfriend or boyhood or child* or girl? or infant* or juvenil* or kid? or minors or minors* or neonat* or neonat* or newborn* or new-born* or paediatric* or peadiatric* or pediatric* or perinat* or preschool* or puber* or pubescen* or school* or teen* or toddler? or underage? or under-age? or youth*)
S3 MH “Postoperative Complications”) OR (((post operative or post-operative or post-surg* or post surg* or surg*) N2 complication*))
S4 (MH “Length of Stay”) OR (hospital stay or hospital stays or “length of stay” or stay, hospital or stay length or stay lengths or stays, hospital)
S5 (MH “Readmission”) OR (30 day readmission or readmission or rehospitalization* or rehospitalization*)
S6 (MH “Hospital Mortality”) OR (((in-hospital or inhospital or in house or in-house) N2 (death or mortality*))
S7 (MH “Health Facility Costs”) OR (((hospitalization or hospitalization or hospital) N2 cost*))
S8 (MH “Treatment Outcomes”)

 © 2025. The Author(s).

Supplementary Table S4 (*Continued*)

S9 S3 OR S4 OR S5 OR S6 OR S7 OR S8
S10 S1 AND S2 AND S9
S11 case report
S12 S10 NOT S11

Supplementary Table S5 Web of Science (Clarivate Analytics interface), 2000 to April 5, 2024

TS = enhanced NEAR/3 recovery (Topic)
AND
TS = (adolescent or *child or *infant or infant disease* or childhood disease* or adolescent* or babies or baby or boy? or boyhood or child* or girl? or infant* or juvenil* or kid? or minors or minors* or neonat* or neonat* or newborn* or new-born* or paediatric* or peadiatric* or pediatric* or perinat* or preschool* or puber* or pubescen* or school* or teen* or toddler? or underage? or under-age? or youth*) and Preprint Citation Index
AND
TS = ((post operative complication* or post-operative complication* or post-surgery complication* or post surgical complication* or surgery complication*or surgical complication*)) OR
TS = ("Length of Stay" or (hospital stay or hospital stays or "length of stay" or stay, hospital or stay length or stay lengths or stays, hospital)) and Preprint Citation Index OR TS= (Patient Readmission or 30-day readmission or readmission or rehospitalization* or rehospitalization) and Preprint Citation Index OR TS = (Hospital Mortality or (in hospital NEAR/2 (death or mortality*))) or TS= (in house NEAR/2 (death or Mortalit*)) and Preprint Citation Index OR TS = (hospitalization cost or hospitalization cost or hospital cost) and Preprint Citation Index OR TS = (treatment outcome) and Preprint Citation Index (Exclude – Database) exclude case report

Supplementary Table S6 Elsevier interface, 2000 to April 5, 2024

((TITLE-ABS-KEY (treatment AND outcome))<br>OR (TITLE-ABS-KEY ((hospitalization AND cost OR hospitalization AND cost OR hospital AND cost)))<br>OR (TITLE-ABS-KEY ((inhouse OR in-house OR hospital OR in-hospital) W/2 (death OR mortality*))) OR (TITLE-ABS-KEY (hospital AND mortality))<br>OR (TITLE-ABS-KEY (patient AND readmission OR 30 day AND readmission OR readmission OR rehospitalization OR rehospitalization))<br>OR (TITLE-ABS-KEY ("length of stay" OR hospital AND stay OR hospital AND stays OR length AND of AND stay OR stay AND length OR stay AND lengths))<br>OR (TITLE-ABS-KEY ("post-operative complication" OR "post-operative complication" OR "post-surgery complication" OR "post surgical complication" OR "surgery complication" OR "surgical complication"))<br>OR (TITLE-ABS-KEY (post AND operative AND complication* OR post-operative AND complication* OR post-surgery AND complication* OR post AND surgical AND complication* OR surgery AND complication*or AND surgical AND complication*)))<br>AND (TITLE-ABS-KEY (adolescent OR *child OR *infant OR infant AND disease* OR childhood AND disease* OR adolescen* OR babies OR baby OR boy? OR boyfriend OR boyhood OR child* OR girl? OR infant* OR juvenile* OR kid? OR minors OR minors* OR neonat* OR neonat* OR newborn* OR new-born* OR pediatric* OR pediatric* OR pediatric* OR perinat* OR preschool* OR puber* OR pubescen* OR school* OR teen* OR toddler? OR underage? OR under-age? OR youth*))<br>AND (TITLE-ABS-KEY ((enhanced W/3 recovery)))

 © 2025. The Author(s).
